# Selections and Abstracts

**Published:** 1908-04

**Authors:** 


### SELECTIONS AND ABSTRACTS.

### REPORT OF THE BOARD OF HEALTH.

To the Honorable Board of Health:

Gentlemen: I have the honor to submit the following report of the work done in the laboratory of hygiene during the year 1907.

### DIPHTHERIA EXAMINATIONS.

During the year 771 cultures were examined for diphtheria, as follows:

| | Primary. | Secondary. |
|---|---|---|
| Positive diphtheria cultures | 94 | 113 |
| Negative diphtheria cultures | 193 | 89 |
| Doubtful diphtheria cultures | 60 | ... |
| Total cultures ...... ... ... ..... ... ... ... ... | | ...549 |

### SPUTUM.

| | Positive. | Negative. |
|---|---|---|
| Sputum examination for T.B. | 38 | 36 |
| Total examinations for T. B. | | 74 |

### MILK EXAMINED.

| | |
|---|---|
| Number samples analyzed (obtained by inspector...... | ..2,132 |
| Number samples (sent in)...... .... ........ .... ...... | 29 |
| Total number analyzed ...... ...... ...... ...... | ..2,161 |

### DIPHTHERIA.

The report shows that there has been a marked decrease in the number of cases of diphtheria during 1907 over previous years. This decrease appears to be due, to a certain extent, to the fact that physicians now send in cultures from every case of suspicious sore throat. This is evidenced by the 193 cultures which were sent from suspicious cases, but which proved negative. Altogether, this indicates that we have almost complete control of all cases of diphtheria which may develop in this city.

### TUBERCULOSIS.

By reason of the increased appropriation last July, we were able to take up the free examination of sputum for the physicians throughout the city. Previously we had no registration or control of tuberculosis. These examinations were taken up so as to insure accurate statistics for the disease, and with a view to possible future education of the unfortunate individuals as to control the spread of the disease. From July 1 until December 31, 74 specimens of sputum were examined in the laboratory, 38 of these being positive and 36 negative.

### MILK.

During January 1907, we submitted to the board of health, the milk ordinance which had been drafted with a view to improving the health of the city through its milk supply, This ordinance, after being adopted by your board, was submitted to the council, and after considerable opposition, was finally adopted. This ordinance was somewhat radical and some of the requirements were entirely new to the dairymen. Our investigations show that much of our trouble was due to ignorance and carelessness. In enforcing the ordinance, we have endeavored not to work a hardship, but have proceeded along the lines of education. The Inspectors have cooperated in the enforcement of the new ordinance, and the improvement in the milk supply is surprising, and we anticipated even greater improvements during the present year.

We are glad to report that the Pasteurizing plant, whose milk was found to be dangerous, was forced to discontinue operations, and now only fresh milk is sold in the city.

We have actively enforced that part of the ordinance which applies to the selling of cream, and all cream sold in the city comes up to the required 20 percent. butter fats. This has forced out of business the unscrupulous people who have been selling adulterated cream. In this connection we would call attention to the requirements for ice cream to contain 14 percent. butter fats. We suggest that this be changed to read 10 percent. butter fats for fruit ice cream, and 12 percent. butter fats for plain ice cream.

### MOSQUITOES.

During the summer of 1907 the mosquito ordinance was actively enforced by Dr. Kennedy, and excellent results followed the employment of inspectors to prevent the development of mosquitoes about the residences. The members of the board will recall that this mosquito work was originally undertaken principally for the purpose of exterminating the yellow fever mosquito (stegomyia), and this was practically accomplished by the work of the inspectors. However, there was a small number of culex mosquitoes which developed along the sewer trunks. This was due to the water famine which prevented the flushing of the sewers, and, therefore, allowed the mosquitoes to develop in the stagnant pools.

### WATER SUPPLY.

During the year we made a number of examinations of the city water and found the condition the same as previous years, with the exception of the period of the water famine, during which time the number of bacteria were increased from 1,500 to 2,000 per cubic centimeter. After this time the water again returned to the normal average of 15 to 20 bacteria per centimeter.

As we have mentioned to the board in the past, the water appears to be as good as can be obtained from our present source of supply. However, there is more or less pollution of the water above the point from which the supply is obtained, and, as the population along the river bank increases, this pollution is hardly dangerous at this time, but in the course of time it is liable to present a serious situation. It is urgent, therefore, that steps should be taken towards securing control of the water shed for the city supply. If appropriation can be secured for this purpose it would pay the city to begin this investigation as early as possible.

Respectfully submitted.

CLAUDE A. SMITH, M. D.,
Director of Laboratory of Hygiene.

---

### SOUTH CAROLINA TO IMPROVE HER MEDICAL PRACTICE LAW.

---

At the meeting of the Legislative Committee of the State Medical Association in conjunction with members of the Association from nearly every county in the State, a bill was drawn up looking toward the improvement of the law now existing as to medical practice in this State.

THE BILL.

To amend an act entitled "An act to regulate the practice of medicine in South Carolina, to provide for a State Board of Medical Examiners and to define their duties and powers," approved February 27, 1904.

Section 1. Be it enacted by the General Assembly of the State of South Carolina that an act entitled "An act to regulate the practice of medicine in South Carolina, to provide for a State Board of Medical Examiners and to define their duties and powers," approved February 27, 1904, be, and the same is hereby amended by inserting immediately after Section 5 thereof a section to be known as Section 5a, as follows:

"Section 5a. The said Board of Medical Examiners is hereby authorized and empowered to suspend or revoke subject, on appeal, to revision by the circuit courts of the State, by a majority vote of its total membership, the license of any practicing physician or surgeon qualified under any provision of this act, and whether qualified prior or subsequent to the passage of this act, after due notice and fair opportunity for hearing, upon its being made satisfactorily to appear that the holder thereof is guilty of felony or gross immorality or is addicted to the liquor or drug habit to such a degree as to render him or her unworthy or unfit to practice medicine in this State, or has been convicted in a court of competent jurisdiction of illegal practices. And the said board is further authorized and empowered to administer oaths in the taking of testimony upon any and all matters pertaining to the business or duties of the Board."

Section 2. That said act be, and the same is hereby, further amended by striking out Section 13 of said act and inserting in lieu thereof the following:

"Section 13. It shall be unlawful for any person or persons to practice medicine or surgery or any branch or specialty of the same in this State, who has failed to comply with the provisions of this act, and anyone violating the provisions of this act shall be deemed guilty of a misdemeanor, and for each offense, upon conviction of any court of competent jurisdiction, shall be fined in any sum not less than fifty dollars, nor more than three hundred dollars, or imprisonment in the county jail for a period of not less than thirty, nor more than ninety days, or both, at the discretion of the court; one-half of the said fine to go to the informant, and the other half to the State; Provided, That dentists and mid-wives shall not be subject to the provisions of this section; Provided, further, That the State Board of Medical Examiners shall issue license to osteopaths and homeopaths specifically for the purpose of practicing respectively when the applicant presents a diploma from a duly authorized school of osteopathy or homeopathy and satisfactorily passes examination before the State Board of Medical Examiners on all regular branches upon which applicants for license to practice medicine are examined except materia medica and therapeutics, major surgery and the practice of medicine.

Section 3. All acts and parts of acts inconsistent with this act are hereby repealed.

Section 4. This act shall go into effect immediately upon its approval by the governor.—*Medical Journal of South Carolina, Feb.* '08.

---

### TRUTH AND THE AMOUNT OF YOUR PRACTICE.

### WHY DOES THE DOCTOR EXAGGERATE HIS STORY OF SUCCESS.

Oliver Wendell Holmes, the gentlest of philosophers of the medical guild, contended that horses had "all the forms of moral excellence, except truth, which does not agree with any kind of horseflesh." We, who are neither gentle nor philosophers, give it as our opinion that the young doctors may, in some particulars, be classed with Holmes' horses. They may present all forms of moral excellence, except truth as to the extent and size of their practices, which does not seem to agree with any kind of young-doctor-flesh. The young doctor, as we have found him and as we have personally experienced him, is, if normal and healthy, an exceedingly cheerful liar in regard to his professional success. In setting down this fact, we wish that there could be some other word to use, for "liar" has a harsh and pernicious sound, while the lies of the young doctor are in every way harmless, in that they seldom or never deceive anyone. They are told with the same thoughtlessness which characterizes many of our verbal conventionalities—such as the "my dear sir," with which we address a letter to our enemy, or the "with sentiments of respect," with which we close a communication to one who has not our respect or that of any other person under heaven.

The young doctor who has had the benefit of pure and orthodox training, sometimes couches his lies with vague and indefinite terms and therewith soothes his delicate conscience. If asked how much he has made in practice within a fortnight, he replies that he has booked so much, his system of booking being peculiarly his own.

A hard-headed business man, who went on an excursion with a number of young doctors and who was made the victim of their numerous professions of prosperity, declared to me that medical men must save more money than those of any other vocation, inasmuch as all of those who had talked to him were making a great deal, while none of them spent much money and all owed as much as they could without bringing about a condition of commercial unrest.

I have gotten so that I am greatly refreshed to find a young physician who is not, by his own confession, grievously overworked. As a rule, if I ask a young doctor how he feels, he yawns a forced yawn and tells me that he will be feeling all right if he can get in a night's sleep without being disturbed by his many over-exacting patients. Yet, as he walks awayI am always inclined to believe that I detect a peculiar shininess of the seat of his trousers and a baginess about the knees which my peculiar diagnostic skill and long experience, impress me as being due to no other etiologic factor than sitting for prolonged periods in a comfortable office chair.

And why, ye scientific investigators, is the young doctor so inveterate a liar on this one subject? Are we to account for it as we do for the whistle in which one always indulges when he passes a cemetery on a dark night—merely to keep up the spirits on a very desolate and lonely occasion? Or, on the other hand, does he indulge in lies merely for the reason that a dog has fleas—

HY-
DRO-
LEINE

AN EMULSION
OF COD-LIVER OIL
OF PROVED
RELIABILITY

A Sample of
Hydroleine
with literature
will be sent gratis, on request.

Pure, fresh cod-liver oil—thoroughly emulsified, unusually palatable, extremely digestible and devoid of medicinal admixtures.

Sold by druggists

THE CHARLES N. CRITTENTON CO.
115 FULTON ST., NEW YORK

merely to show that he is a dog—simply to show that he is a young doctor.

A friend of mine remarked to me that young Doctor Blank was out six nights in the week in his large obstetrical practice—she knew it because he told her so. And this friend of mine added, "I did not know there was that much charity obstetrical practice in this community." Which merely indicates, my dear young doctors, that your stories of exploit are received by the simple minded layman with an inward and indulgent smile and not with the same degree of seriousness with which you deliver them.

That the young doctor's lies are not told with any intent to deceive, however, is indicated by the fact that they are told to older and other practitioners quite as freely as the uninitiated layman. A physician of the vintage of 1907 recently wrote me: "I should be very glad to submit a report of this case. The condition is an exceedingly rare one. I recall but four like it in my entire experience!"—an experience which has extended from July, 1907, to January, 1908!

But why protest against the thing? With young doctors it is very much like the assumption of great dignity—a silly puerile, but very harmless thing which is all right if outgrown early. As we grow to be men, however, pomposity and prevarications of prosperity become exceedingly transparent evidences of little mentality. When we have become men we should have put away childish things.—*Chicago Clinic and Pure Water Journal, March, 1908.*

---

### THE BEST HE KNEW.

---

Gladstone, a Jamaican negro, was assistant to a district physician in the Canal Zone, and, being rather poor in his Latin, the bottles had been numbered for his benefit. One day a Spanish laborer came in for medicine, and the doctor told his worthy assistant to give him two pills out of number six. After he had gone the doctor asked:

"Gladstone, did you give the man a dose of number six?"

"Oh, no, sah, Doctor; number six war finished, so I just give him one pill out of numbah foah and one out of numbah two."—*March Lippincott's.*

---

Billy, aged twelve, took part the other day in a debate on imperialism. His opponent, in rebuttal, made a point by quoting the definition of empire from the Century Dictionary. Billy, nothing daunted, with all the air of Patrick Henry himself, rose up and said:

"It's all right for my opponent to quote from the dictionary, but as for me, I rely on *the facts!"*

---

His clothes were spotted with dirt and grease, but a bright bunch of flowers adorned the lapel of his coat.

"What do you think of this?" he asked, proudly tapping his bouquet. "Where do you think I got it?"

"Don't know," admitted his friend, "unless—Why, maybe it grew there."—*January Everybody's*

